# Mouthpiece ventilation in the management of dyspnea: A single-arm pilot study

**DOI:** 10.1177/0269216320935003

**Published:** 2020-06-24

**Authors:** Juho T Lehto, Sirpa Leivo-Korpela, Tarja Korhonen, Heidi A Rantala, Hanna Raunio, Tiina Lyly-Yrjänäinen, Lauri Lehtimäki

**Affiliations:** 1 Faculty of Medicine and Health Technology, Tampere University, Tampere, Finland; 2 Palliative Care Centre and Tays Cancer Centre, Department of Oncology, Tampere University Hospital, Tampere, Finland; 3 Department of Respiratory Medicine, Tampere University Hospital, Tampere, Finland; 4 Pirkanmaa Hospice, Palliative Care Centre, Tampere University Hospital, Tampere, Finland

**Keywords:** Dyspnea, noninvasive ventilation, mouthpiece ventilation, palliative care, palliative medicine

## Abstract

**Background:**

Noninvasive ventilation may relieve dyspnea in advanced diseases, but noninvasive ventilation through mouthpiece has not been tested in palliative care.

**Aim:**

To assess the feasibility of mouthpiece ventilation in relieving dyspnea among patients with advanced disease.

**Design:**

In this prospective single-arm pilot study, the change in dyspnea by mouthpiece ventilation was measured with numeric rating scale (0–10) and 100-mm visual analogue scale. Overall, benefit and adverse events of the therapy were also assessed.

**Setting/participants:**

Twenty-two patients with an advanced disease and dyspnea from the Tampere University Hospital or Pirkanmaa Hospice were treated with mouthpiece ventilation. The patients used mouthpiece ventilation as long as they preferred, but for a minimum of 5 min.

**Results:**

After the treatment period lasting a median of 13.5 min, mean decrease in dyspnea was −1.1 (95 % confidence interval = −2.2 to −0.1, *p* = 0.034) on numeric rating scale and −11.8 mm (95 % confidence interval = −19.9 to −3.7, *p* = 0.006) on visual analogue scale. Nonetheless, there was a high variability in this effect between individual patients. About half of the patients found mouthpiece ventilation beneficial. No serious adverse events occurred, but dry mouth was the most common adverse event. Anxiety did not increase with mouthpiece ventilation.

**Conclusion:**

Mouthpiece ventilation is feasible and may relieve dyspnea in some patients with an advanced disease. Further studies are needed, and these might concentrate on stable patients in early palliative care. Before initiation, this study was registered at clinicaltrials.gov (study no. NCT03012737).

**What is already known about the topic?**

- Noninvasive ventilation relieves dyspnea, but mouthpiece ventilation has not been tested in palliative care.

**What this paper adds?**

- Mouthpiece ventilation is feasible and safe in patients with advanced diseases.
- Dyspnea seems to be relieved by mouthpiece ventilation in many of these patients.
- The efficacy and compliance of the therapy differs markedly between individual patients.

**Implications for practice, theory or policy**

- The role of mouthpiece ventilation should be confirmed in further controlled studies including stable patients in early palliative care.

## Introduction

Dyspnea is a common and distressing symptom in patients with advanced diseases.^1,2^ Management of dyspnea is challenging and new therapies are urgently needed.^3,4^ In addition to hand held fan and some breathing techniques,^3,5^ noninvasive ventilation has been shown to reduce dyspnea in patients with end-stage diseases, but the use of a face mask may cause distress and pain.^6–10^ Mouthpiece ventilation is an option to deliver noninvasive ventilation via an open-circuit mouthpiece, and this might offer advantages such as maintaining the ability to speak and eat.^11^ However, to our knowledge, mouthpiece ventilation has not been studied in the context of palliative care. The aim of this pilot study was to assess the feasibility of mouthpiece ventilation in relieving dyspnea among patients with an advanced disease.

## Material and methods

This was a single-arm pilot study on the management of refractive dyspnea with noninvasive ventilation via mouthpiece. The primary end-point was a change in the intensity of dyspnea measured by numeric rating scale after the first treatment period on mouthpiece ventilation.

### Patients

The patients were recruited between January 2017 and April 2019 from the Tampere University Hospital or Pirkanmaa Hospice (Tampere) and followed up until the end of November 2019. Inclusion criteria were an incurable and advanced life-limiting disease, dyspnea ⩾4 on numeric rating scale despite the other therapies of dyspnea provided by the attending physician, age ⩾18 years, capability to give written informed consent and physician’s decision to withhold resuscitation and admission to intensive care unit. Patients with decreased level of consciousness, insufficient co-operation or a treatable cause of dyspnea were excluded.

### Intervention

The patients used mouthpiece ventilation for a minimum of 5 min, but as long as they desired. After this treatment period, the patients were allowed to use the ventilator as they wanted during the next 24 h. All the other treatments of dyspnea were permitted.

**Figure 1.**
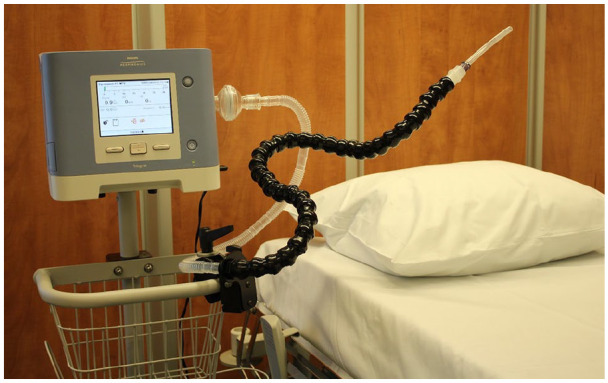
Ventilator with the equipment for the mouthpiece ventilation.

Mouthpiece ventilation was provided using a Trilogy 100^®^ (Philips Respironics, Murrysville, PA, USA) ventilator with an angled or a straw-type mouthpiece (Figure 1). Inspiratory pressure, inspiratory time and rise time were adjusted according to each patient’s preference. The patients were taught to inhale through the mouthpiece and exhale either by taking the mouthpiece out from their mouth or by loosening their lips around the mouthpiece.

### Assessments

Intensity of dyspnea, pain and anxiety were measured by numeric rating scale from 0 (no symptom) to 10 (the worst possible symptom) and visual analogue scale from 0 (no symptom) to 100 mm (the worst possible symptom) before the intervention, after the first treatment period, and for dyspnea also after 5 min on mouthpiece ventilation. Dryness of mouth, accumulation of air into stomach, sense of panic and other possible adverse events were measured by numeric rating scale after the therapy. Serious adverse events leading to death or serious deterioration of the patient were also assessed.^12^ The patients’ opinion on the benefits and compliance of mouthpiece ventilation were asked by using a Likert-type scale ranging from 1 (totally disagree) to 5 (totally agree). Oxygen saturation, breathing frequency and heart rate were measured as well.

### Statistics

This study originally aimed at a higher number of patients, but due to their frailty, we were able to recruit 22 patients during the recruitment period. This sample size provides a statistical power of 84% to detect a decrease of at least 1.0 in dyspnea on numeric rating scale scale with an alpha error of 5% and standard deviation (SD) of 1.5. A paired samples *t*-test was performed to compare continuous variables as most of them were normally distributed. Statistical significance was set as *p* < 0.05. Analyses were performed with IBM SPSS Statistics version 25.0 (IBM Corp, Armonk, NY, USA).

### Ethical considerations

The study was approved by the Ethics Committee of Tampere University Hospital (R16111; 25 August 2016) and all the subjects gave their written informed consent. Before initiation, this study was registered at clinicaltrials.gov (study no. NCT03012737).

## Results

Twenty-two patients were included to the study (Table 1).

**Table 1.** Patient characteristics.

*N*	22	
Age (years), median (range)	75	(27–84)
Female, *n* (%)	13	(59.1)
Survival (days), median (IQR)	34	(16–97)
Diagnoses, *n* (%)		
Lung cancer	12	(54.5)
Gastrointestinal cancer	3	(13.6)
Lymphoma	2	(9.1)
Breast cancer	1	(4.5)
Renal cancer	1	(4.5)
Sarcoma	1	(4.5)
Spinocellular cancer	1	(4.5)
Bronchiectasis	1	(4.5)
Intrathoracic spreading of cancer	17	(77.3)
Place of treatment, *n* (%)		
Hospital ward	14	(64)
Hospice	8	(36)
Discharged to home, *n* (%)	9	(41)

IQR: interquartile range.

All the patients had dyspnea intensity of ⩾4 on numeric rating scale during inclusion, but with four patients, the dyspnea score decreased under 4 before the initiation of mouthpiece ventilation. The patients used mouthpiece ventilation for a median of 13.5 min (interquartile range (IQR): 9.25–20.0) in the first treatment period. After the first treatment period, 19 (86 %) patients used mouthpiece ventilation with a total median time of 35 min (IQR: 16–45) during 24 h (one-time period missed for technical reason).

### Change in dyspnea

**Figure 2.**
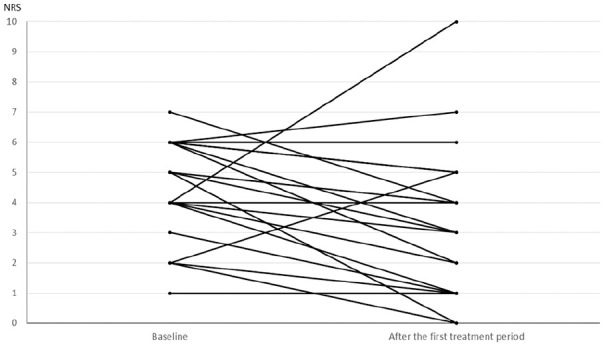
Change in the severity of dyspnea on numeric rating scale after the first treatment period on mouthpiece ventilation.

Change in dyspnea in individual patients after mouthpiece ventilation is shown in Figure 2. Mean decrease in dyspnea after 5 min was −1.3 (95 % confidence interval (CI) = −2.2 to −0.3, *p* = 0.013) on numeric rating scale and −11.6 mm (95 % CI = −19.7 to −3.5, *p* = 0.007) on visual analogue scale, while the corresponding changes after the first treatment period were −1.1 (95 % CI = −2.2 to −0.1, *p* = 0.034) and −11.8 mm (95 % CI = −19.9 to −3.7, *p* = 0.006). One patient with severe anxiety (8 on numeric rating scale) and two patients with increasing mucus in airways reported dyspnea increment after mouthpiece ventilation.

### Overall benefit and adverse events

Severity of pain or anxiety did not significantly change during the treatment. Adverse events and patients’ opinions concerning the mouthpiece ventilation are presented in Table 2. About half of the patients found mouthpiece ventilation beneficial and might use it again. No serious adverse events occurred. Mean decrease in breathing frequency was −2.1 breaths per minute (95 % CI = − 0.6 to −3.6, *p* = 0.01) after 5 min and −1.2 (95 % CI = −0.6 to −3.6, *p* = 0.22) after the first treatment period. There was no significant change in oxygen saturation or heart rate.

**Table 2.** Proportion of the patients reporting adverse events and agreeing partly or completely with the opinions concerning the mouthpiece ventilation after the first treatment period.

	*n* (%)	Numeric rating scale, median (range)^a^
Adverse events after mouthpiece ventilation		
Dry mouth	18 (82)	3 (1–10)
Air accumulation into stomach	7 (32)	2 (1–6)
Sense of panic	3 (14)	2 (1–3)
Headache	2 (9)	(3–4)
Increased respiratory secretions	2 (9)	Not available
Opinions on mouthpiece ventilation		
Mouthpiece ventilation relieved my dyspnea	12 (55)	
Mouthpiece ventilation was beneficial for me	10 (45)	
I complied well with mouthpiece ventilation	16 (73)	
Mouthpiece ventilation was unpleasant	3 (14)	
I would like to use mouthpiece ventilation again for my dyspnea	14 (64)	

a Only patients with numeric rating scale >0 included.

## Discussion

### Main findings of the study

In our pilot study, most of the patients with advanced disease and dyspnea complied with mouthpiece ventilation without serious adverse events. On average, dyspnea was slightly relieved, but there was notable variation between the patients.

The mean decrease in dyspnea by mouthpiece ventilation was statistically significant and about 1.2 points on numeric rating scale. However, due to our uncontrolled study design, the relief of dyspnea might be related to care effect rather than mouthpiece ventilation. Thus, our preliminary result has to be interpreted with caution and confirmed in further controlled studies.

As this is the first study on mouthpiece ventilation in palliative care, we are not able to compare our results directly to previous studies. In a study by Nava et al. on patients with advanced cancer, treatment with mask noninvasive ventilation led to an average reduction of dyspnea of about 2–3 points on a Borg scale.^7^ This can be considered larger effect than in our study, but the method and the patient group were different.^7^ Patients with neuromuscular disease using mouthpiece ventilation have experienced relief of dyspnea, but the quantitative reduction in dyspnea has not been reported.^13^ Airflow through a fan or nasal cannula are shown to relieve dyspnea with a comparable effect to our results on mouthpiece ventilation.^5,14^

The most common adverse events during mouthpiece ventilation were dry mouth and accumulation of air in stomach, which are known side effects of noninvasive ventilation with a mask as well.^13,15^ In the previous studies, noninvasive ventilation with a mouthpiece is reported less painful compared with noninvasive ventilation with a mask.^13^ None of our patients reported pain after mouthpiece ventilation and anxiety was not increased. Some adverse events of noninvasive ventilation with a mask, like vomiting and aspiration, did not occur in our study.^10,15^

Although most of our patients complied well with mouthpiece ventilation, some of our frail patients (median survival of about 1 month) had difficulties in adapting to the therapy, which probably explained quite short ventilator using times. One of our patients with severe anxiety and two with increasing respiratory secretions found mouthpiece ventilation unpleasant and reported even increase in dyspnea highlighting the importance of careful patient selection. Usage of mouthpiece ventilation requires skilled guidance from personnel and co-operation from the patient.^16,17^

### Strengths and limitations of the study

Strength of our study was the description of a novel treatment modality for dyspnea and systematic assessment of possible adverse events. The relatively small patient population and the lack of a control group limit the conclusions regarding possible benefits of the treatment. Finally, we did not distinguish chronic and episodic dyspnea in our study.^18,19^

## Conclusion

Mouthpiece ventilation is feasible and safe among patients with advanced disease and it might be a beneficial treatment option for some patients in relieving dyspnea. However, frail patients may be unable to use mouthpiece ventilation long enough to receive optimal benefit. Further controlled studies are needed to determine the efficacy of mouthpiece ventilation and to compare it to other treatment options. These studies might include patients in early palliative care with chronic breathlessness.
